# Power Efficient Machine Learning Models Deployment on Edge IoT Devices

**DOI:** 10.3390/s23031595

**Published:** 2023-02-01

**Authors:** Anastasios Fanariotis, Theofanis Orphanoudakis, Konstantinos Kotrotsios, Vassilis Fotopoulos, George Keramidas, Panagiotis Karkazis

**Affiliations:** 1 Digital Systems and Media Computing Lab, School of Sciences and Technology, Hellenic Open University, 26334 Patras, Greece; 2 Department of Informatics, Aristotle University of Thessaloniki, 54124 Thessaloniki, Greece; 3 Department of Informatics and Computer Engineering, University of West Attica, 12243 Athens, Greece

**Keywords:** machine learning, power efficiency, edge computing, autonomous systems, neural networks compression

## Abstract

Computing has undergone a significant transformation over the past two decades, shifting from a machine-based approach to a human-centric, virtually invisible service known as ubiquitous or pervasive computing. This change has been achieved by incorporating small embedded devices into a larger computational system, connected through networking and referred to as edge devices. When these devices are also connected to the Internet, they are generally named Internet-of-Thing (IoT) devices. Developing Machine Learning (ML) algorithms on these types of devices allows them to provide Artificial Intelligence (AI) inference functions such as computer vision, pattern recognition, etc. However, this capability is severely limited by the device’s resource scarcity. Embedded devices have limited computational and power resources available while they must maintain a high degree of autonomy. While there are several published studies that address the computational weakness of these small systems-mostly through optimization and compression of neural networks- they often neglect the power consumption and efficiency implications of these techniques. This study presents power efficiency experimental results from the application of well-known and proven optimization methods using a set of well-known ML models. The results are presented in a meaningful manner considering the “real world” functionality of devices and the provided results are compared with the basic “idle” power consumption of each of the selected systems. Two different systems with completely different architectures and capabilities were used providing us with results that led to interesting conclusions related to the power efficiency of each architecture.

## 1. Introduction

Ubiquitous or pervasive computing [1], the 3rd Era of computing (or the 3rd technological wave) [2] has already dawned. The average human today has at his or her disposal a large number of computational resources without even noticing their existence. Despite mobile devices, smartphones, and wearable devices, a vast amount of similar pervasive computing systems are hidden from us working in the periphery of the human sensing range. These kinds of smart human-centric services are running inside vehicles in the form of numerous Automotive Driving Assisting Sub-systems (ADAS) [3], in smart traffic light control systems within a smart city [4], or in smart home environments.

Today, a human is constantly surrounded by numerous hidden embedded systems. Some of them are inside devices and are designed to require user input to function correctly but the newer/advanced ones are totally invisible context-aware systems that require no user input and operate autonomously minimizing users’ attention towards them. Some of them have direct access to the Internet or form a Machine to Machine (M2M) network between them, and in any case, they execute some kind of smart algorithm to provide human-centric services. IoT systems tend to outsource any computationally heavy workloads, through their network, to bigger, powerful servers forming in such a way, a-big-centralized system; however, centralized systems face the same challenges of autonomy (relying on a central server to function), privacy (constant data transfer) and a degree of ineffectiveness both regarding network bandwidth as well as device resource usage.

Beyond pure IoT (or extreme edge) systems there are also edge devices. While IBM has stated that Edge devices are significantly more powerful than IoT devices [5] (thus less energy efficient and less power-autonomous) as systems designed around High-power microprocessors or accelerator ASICs and, in this way, it created a new category of extreme edge devices that are similar to IoT devices. In reality, today these categories are defined by the processing unit architecture. A low-power microcontroller architecture is the de-facto choice of a designer for an IoT device as well as for an autonomous small Edge device, as long as low power consumption is a requirement. The “Lower Power” Term is what is constantly changing, with newer architectures achieving better power efficiency. An example is the Cortex M Series from ARM, where the M3 and M4 architectures were considered the state-of-the-art Low power architectures until Cortex M0 and later M0+ architectures were released to the public as low-power devices (Lower than the M3 and M4 counterparts). In this work we focus on the aspect of the processing unit architectures and power efficiency, thus, we consider that an edge system should also be power efficient to be autonomous. In many cases, this aspect makes these systems indistinguishable-at least on the hardware side-from their IoT counterparts with the only difference being that they process the data before exporting it as a service. Of course, this increases their autonomy since there is no functional need for a server, albeit with the added cost of increased power consumption. It also increases their networking effectiveness since the device only needs to transmit the results of a service or nothing if it provides the service locally and it strengthens its security/privacy abilities since no raw data (e.g., video feeds or other sensor data) are transmitted over the network.

Autonomy is the key concept in this case. An autonomous smart device is more secure, efficient, and flexible. One significant aspect of a device’s autonomy is its power autonomy which is directly correlated with the device’s power efficiency. While there are numerous methods that affect a microcontroller’s power efficiency, and although the microcontrollers manufacturers have turned their focus on developing efficient designs and toolchains to exploit them, the issue of running ML models in small power-efficient microcontroller-based systems is still largely unexplored. A significant number of studies on GPU-based systems, or embedded systems based on power-hungry ASICs but there is little information available on how ML models-based compression or optimization method affects power usage in systems with restricted computational resources.

This paper presents the results of power efficiency measurements of selected ML models, both compressed and uncompressed, for different microcontroller architectures. The microcontroller selection was made according to architecture popularity on today’s devices albeit limited in the devices with relatively large -for a microcontroller- number of resources (SRAM & Flash-memory size) to increase the capability to fit uncompressed models to the device. The selection of the ML models was done from a group of proven models limited only by their size since they need to be able to fit and run with the available storage and memory for each system.

## 2. Related Work

Since the first ML models were ported to embedded systems a lot of research has been done regarding the optimization of such models to run efficiently on these smaller systems. However, most of the published works consider such systems either mobile devices, centering mostly around smartphones as a generic ML platform, Tensor Processing Units (TPUs) as discrete embedded systems employing ASICs or FPGAs or other small development embedded systems such as raspberry Pi while seldom taking into account microcontroller-based systems. In [6] the authors review the challenges in using ML on embedded devices i.e., the computational and memory-intensive nature of many ML algorithms, such as hidden Markov models (HMMs), k-nearest neighbors (k-NNs), support vector machines (SVMs), Gaussian mixture models (GMMs), and deep neural networks (DNNs). To overcome these limitations, specialized optimization techniques are often used to reduce the footprint of the implementation of these algorithms in ways that they can fit into the limited hardware resources available on these devices.

Specialized ML frameworks are commonly used to port ML algorithms to resource-limited hardware platforms such as MCUs, mobile devices, and accelerators. However, this can also introduce challenges, such as the need for hardware-oriented and algorithm-oriented optimization schemes to address bottlenecks in performance. Specific techniques such as model pruning and data quantization [6] can be used to optimize ML algorithms for these types of devices.

There are many potential applications for embedded ML, including systems such as edge computing and sensor networks. However, there is also a number of open research issues and challenges in this field, such as the trade-off between model accuracy and resource constraints, and the need for robustness and security in these systems.

Overall, the study presented in [6] provided an overview of the landscape of embedded ML, including common algorithms, optimization techniques, hardware platforms, challenges, open research issues, and potential applications.

A large amount of research has been done presenting methods that increase the power efficiency of ML algorithms running on embedded systems, such as event-based inference methods that induce a “smart triggering” of the power-hungry inference algorithm under certain criteria only. In the machine-vision application presented in [7], the authors used event-based vision sensors that encode scenes using streams of events that represent local pixel-wise brightness changes, rather than full image frames. This resulted in a sparse, energy-efficient representation of the scene, as well as low inference latency. The authors of this article proposed a hybrid architecture for end-to-end training of DNNs for event-based pattern recognition and object detection. The architecture combines a spiking neural network (SNN) backbone for efficient event-based feature extraction, with a classical analog neural network (ANN) to solve synchronous tasks. This was made possible by a combination of standard backpropagation training with surrogate gradient training. The use of hybrid SNN-ANN architectures allowed for training without additional conversion steps, resulting in highly accurate networks that are more computationally efficient than their ANN counterparts. The authors demonstrated their approach to event-based classification and object detection datasets, showing that only the ANN heads need to be adapted to the specific tasks, without requiring any conversion of the event-based input. Since ANNs and SNNs require different hardware architectures to maximize their efficiency, the authors envisioned that the SNN backbone and ANN head could be executed on different processing units, with the necessary communication between the two parts. Overall, hybrid networks are a promising approach for advancing ML for event-based vision, without sacrificing or even increasing efficiency.

Other techniques define step-based methods of optimization and compression of ML models to decrease their memory and storage footprint which inadvertently decreases power consumption as the running time for each inference decreases. Authors in [8] showed deep compression as a technique that can reduce the storage requirements of neural networks by up to 49× without affecting their accuracy. This is achieved by applying three different techniques: pruning, quantization, and Huffman coding. Pruning involves removing unnecessary connections in the network, reducing the number of connections by 9× to 13×. Quantization then reduces the number of bits needed to represent each connection from 32 to 5. Finally, Huffman coding is applied to further reduce the size of the network. This technique has been proved effective on the ImageNet dataset, where it reduced the size of AlexNet by 35× and the size of VGG-16 by 49×, with no loss of accuracy. It can be beneficial for deployment on embedded systems with limited hardware resources, as well as for mobile applications where application size and download bandwidth are constrained. Additionally, this approach provides reduced latency and improved energy efficiency when benchmarked on CPU, GPU, and mobile GPU.

In the work presented in [9], the authors have proposed the One-shot Pruning-Quantization (OPQ) method as a network compression method that aims to reduce the size of large DNNs for deployment on resource-constrained hardware platforms such as smartphones. OPQ differs from other network compression methods in that it analytically solves the compression allocation of each layer without the need for iterative or manual optimization. This makes the method more efficient and easier to use. OPQ also introduces a unified channel-wise quantization method that uses a common codebook for all channels in a layer, which can further improve the efficiency of the method without sacrificing accuracy. Experiments on ImageNet with different DNN architectures show that OPQ achieves significantly higher compression rates than other methods while maintaining high accuracy. In the same study, authors acknowledged that Neural Network (NN) compression is an important area of research and development because it can make deep learning models more efficient and practical for deployment on resource-constrained devices, such as smartphones, and other edge devices. By reducing the memory footprint, computational cost, and power consumption of DNNs, it becomes possible to use these models in applications where this was previously impractical due to their resource requirements. This can enable the use of deep learning in a wider range of applications, including computer vision, speech and audio processing, natural language processing, and recommender systems.

In fact, reviewing the literature on these step/pipelined methods we concluded that as the models reduced, the requirements in terms of processing time per inference and the overall power consumption also decreased. Some methods proposed post-deployment updates for edge devices with various compression methods like those presented in [10]. The authors acknowledged that the use of deep learning models on edge devices, such as smartphones and other portable devices, is an active area of research and development. These devices often have limited memory, processing power, and battery life, which can make it challenging to deploy deep-learning models on them. Neural network compression is one approach that can help overcome these limitations by reducing the memory footprint and computational cost of deep learning models, while often maintaining a similar level of accuracy. This can make it possible to deploy deep learning models on edge devices and enable their use in a wider range of applications. The results of this study, again, lead to power efficiency improvements with minimal to zero requirements of retraining since the initial model retains its original “knowledge”.

In one of the works [11] the authors proposed methods that are targeting microcontroller-based systems, producing small-sized, fast running low power ML models. This study presented a solution to the challenges faced by traditional approaches to using ML in IoT systems. By using an end-to-end optimization sequence, the authors were able to create small, low-latency, low-power models that can be executed on resource-constrained hardware. The experimental results show that these optimized models were more accurate and faster than traditional models, making them a promising solution for IoT applications.

Finally, in [12] the authors focused on AI at the edge referring to the use of AI algorithms and technologies in edge devices, such as sensors, cameras, and other IoT devices, to process data locally, rather than relying on the cloud. This allows for low-latency processing, improved energy and spectrum efficiency, and enhanced security. However, performing sophisticated AI algorithms in resource-constrained edge devices can be challenging. To overcome this, low-power chips and system-level frameworks are needed to distribute resources and tasks along the edge-cloud continuum. Fog computing, which enables processing at the edge while still allowing for interaction with the cloud, can also play a role in bridging the gap between edge devices and the cloud in AI applications. By leveraging the advantages of both edge and cloud computing, AI at the edge can enable a wide range of applications, from autonomous systems and human-machine interactions to IoT and beyond. Some comparisons on the results of this study were found to be meaningful power measurements over different hardware architectures and ML models, albeit the results were not presented in a uniform way, mixing overall power consumption with per-inference and per-instruction results.

## 3. System Setup and Methodology

**Figure 1:**
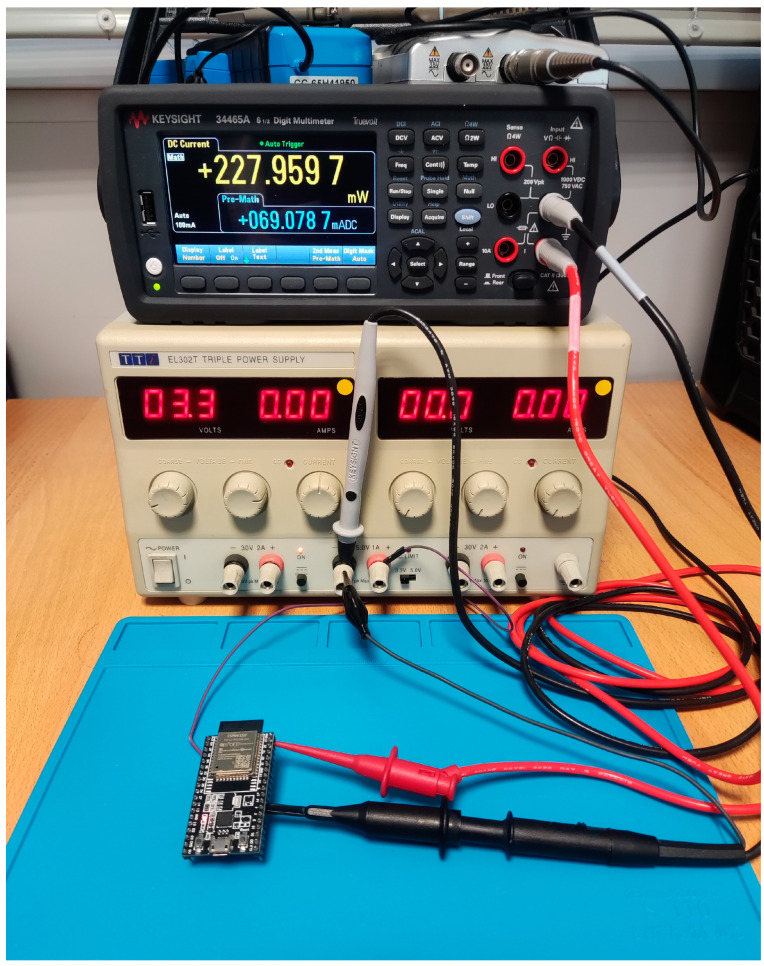
Keysight 34465A DMM and Bench PSU.

To get accurate results, we used a set of instruments that include, a lab-grade Digital Multimeter (DMM) with external triggering support and a bench Power Supply Unit (PSU) that can provide a steady voltage of 3.3 Volts (Figure 1). An additional accurate multimeter was also used to verify the exact voltage measurement when MCU was under load. As IoT/Edge systems we selected two development boards, one board from Espressif (Shanghai, China) with a Dual Tensilica Core as it is widely used in IoT systems, and another one from ST Microelectronics (Plan-les-Ouates, Geneva, Switzerland) based on an ARM Cortex MCU. Both boards were set up and/or modified for direct voltage feed at 3.3 Volt to minimize any power loss from Voltage Regulators. We also disabled any onboard LEDs to minimize power loss from them.

### 3.1. Digital Multimeter & Power Measurements Setup

As a DMM we selected the Keysight 34465A. This is a digital multimeter capable of performing a variety of measurements. It is a highly accurate and reliable instrument that is commonly used in a wide range of applications, from electronic design and manufacturing to field service and laboratory testing. The 34465A is known for its ease of use and ability to measure a wide range of signals with a high degree of precision. It is capable of making measurements with an accuracy of up to 0.0035% + 3 counts, depending on the range and function being used. This level of accuracy is achieved through the use of advanced digital signal processing techniques. This multimeter also includes various built-in self-calibration and self-diagnostic functions that help to maintain its accuracy over time. This device also can be triggered externally, which allows the user to synchronize the measurement with an external event or signal. This can be useful in a number of applications, such as when the multimeter is being used as part of a data acquisition system or when precise timing is required for the measurement. Additionally, it offers a number of math functions that can be used to perform calculations on the measured data. These functions include basic arithmetic operations (addition, subtraction, multiplication, and division), as well as more advanced operations such as square root, square, absolute value, and logarithm. The multimeter also allows the user to define and store up to 10 custom math functions, which can be used to perform more complex calculations or to apply custom mathematical transformations to the measured data. To use the math functions, the user can simply select the desired operation from the multimeter’s front panel menu or via the remote interface. The multimeter will then automatically perform the calculation on the measured data and display the result.

Finally, this device can perform data acquisition and storage, which allows the user to capture a series of measurements over time and save them for later analysis. The multimeter includes a built-in data logger that can store up to 10,000 readings, which can be saved in various formats (including CSV, TXT, and BIN) for easy transfer to a computer or other device. The data logger also allows the user to set a variety of parameters, such as the sampling rate and the number of readings to be taken, to customize the acquisition and storage of the data. To use the data logger, the user can simply configure the settings and start the acquisition using the front panel controls or the remote interface. The multimeter will then automatically capture and store the measurements according to the specified settings. The DMM also can create, display, and export trend charts of measurements.

- To perform accurate measurements, we follow the methodology that is presented below: The 3A current port was selected as input
- Trigger mode was selected with external triggering on the negative edge
- The DMM was set on Continuous Acquire Mode
- A delay of 50 uS was set as a measurement delay after triggering
- The sampling rate was set for each model in such a way that it was possible to take at least three samples during the inference time
- Display mode switched to trend chart
- Added a linear math function that converted Current input (mA) to Power Input (mW)

**Figure 2:**
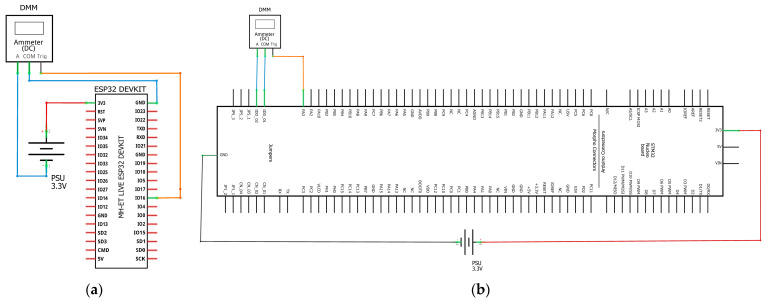
DMM Connection Diagram for Test Board (**a**) ESP32, (**b**) STM32H7.

The PSU was set at 3.3 Volts output, then the DMM Current Port was connected in series with the PSU and the board that was currently under measurement. The voltage was verified with a second DMM to be stable at 3.3 V with the test board running the code. To take sample readings only during the inference time an output pin was selected from the board that was set on low voltage (GRD) just before the inference line of code in the MCU. This pin was connected to the external trigger port of the DMM creating in such a way a negative edge triggering event for it. The connection diagrams are shown below (Figure 2).

The code we developed also provided the total time for each inference by printing it to a text console through an MCU’s serial port output (UART). This value was used both for setting the total samples and sample rate for each measurement in the DMM as well as for calculating the total power needed for each inference to our final results.

Finally, we also measured the power consumption for each board running code in endless loop mode of no operations (NOP). This established a baseline power consumption for each MCU without any user software/firmware running that later permitted us to extract pure inference power consumption results.

### 3.2. Development Boards and MCU Architectures

**Figure 3:**
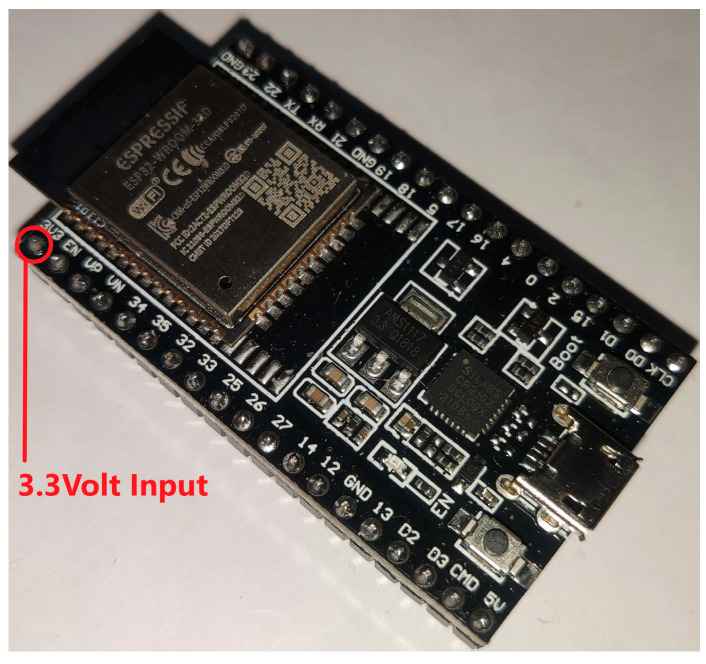
ESP32 Development Board.

The two boards we selected were the ESP32-DevKit by Espressif and STM32H743-nucleo from ST Microelectronics. The ESP32 (Figure 3) is a low-cost, low-power system-on-a-chip microcontroller board with integrated Wi-Fi, dual-mode Bluetooth capabilities, 4 MB of Flash Storage, and 320 KB of SRAM available. It is produced by Espressif Systems, and is commonly used in a wide range of IoT and embedded systems applications. The ESP32 is known for its low power consumption, high performance, and rich set of peripherals and features, which include support for multiple high-speed communication interfaces, touch sensors, and a wide range of embedded peripherals. It is also compatible with a variety of development environments and can be programmed using a variety of languages, such as C, C++, and Python. The architecture of ESP32 is based on a Tensilica Xtensa LX6 dual core that is a high-performance, 32-bit microprocessor developed by Cadence Design Systems and is part of the Xtensa processor family.

**Figure 4:**
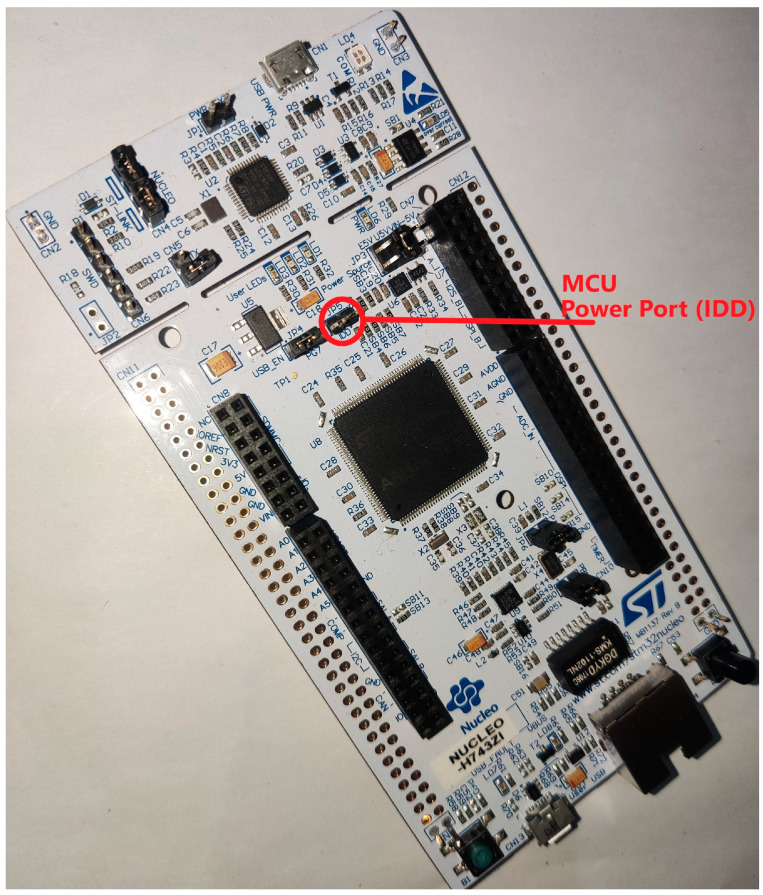
STM32H743-Nucleo Development Board.

The STM32H743-nucleo (Figure 4) is a development board that is based on the STM32H743 microcontroller from ST Microelectronics. The STM32H743 microcontroller is a high-performance device that features a 32-bit Arm Cortex-M7 MCU with a clock frequency of up to 400 MHz, 2 MB of Flash Storage, a sum of 1 MB SRAM and a wide range of peripherals and features, including support for the dual-core operation, a large set of digital and analog peripherals, and a wide range of communication interfaces. The STM32H743-nucleo board provides an easy-to-use platform for evaluating and developing with the STM32H743 microcontroller and includes various onboard components and interfaces that allow the user to quickly connect to and test the capabilities of the microcontroller. The board is compatible with a variety of development environments and can be programmed using a wide range of languages and developer tools.

The ARM Cortex-M7 is a high-performance microcontroller processor core designed by ARM Holdings. The Cortex-M7 core is based on the ARMv7-M architecture and is capable of operating at clock speeds of up to 600 MHz. It offers a number of features that make it well-suited for use in a wide range of applications, including:

- a high-performance, single-cycle 32-bit floating-point unit (FPU) for efficient handling of complex mathematical operations
- advanced interrupt handling capabilities, including support for up to 240 external interrupts and a nested vectored interrupt controller (NVIC)
- high-speed memories, including a Harvard architecture instruction cache, data cache, and tightly-coupled memory (TCM) for fast access to frequently used data
- a large number of peripheral interfaces, including multiple serial communication interfaces, timers, analog-to-digital converters, and digital-to-analog converters
- Each feature may affect the power consumption of ML models in different ways.

As with every other modern microcontroller, these two include a large number of peripherals inside the same package next to the processing core. These peripherals are connected to the core via internal buses with two main functions. The data transfer from and to the core includes all the data toward peripheral setup registers (peripheral control) and the clock feeding/input for each peripheral. Usually, each peripheral requires a specific clock signal that is different from the one that the core runs, and most of them may work over a range of user-selectable clock frequencies that affect their running speed and power consumption. This in turn creates the requirement of using Phased-Lock Loop Devices (PLLs) to manipulate the clock signals for each peripheral.

As soon as the setup register for a peripheral is loaded with the correct configuration data and the clock signal has started, this peripheral’s state is considered active. So, it is obvious that for each active peripheral, an amount of power has to be consumed to feed it with energy and enable the required clock feed. It doesn’t matter which operational status of a peripheral is currently in use; just enabling it contributes to the overall power consumption of the microcontroller. For this reason, the default state for all the peripherals of every modern MCU is disabled, with the exception of the JTAG interface. So, the power consumption of a microcontroller is greatly affected by the enabled peripherals, that in turn are defined by the user or developer of the firmware according to the requirements thus it differs from application to application.

Additionally, every modern microcontroller also supports power management states (or sleep states) that permit them to shut down groups of peripherals and clocks and even freeze/stop the core while retaining some user data to a small part of memory to continue functioning after waking up from a sleep state. Such states reduce the overall power consumption of an MCU to extremely low levels. Again, these sleep states are defined by the developer or user and are different for every application. It is worth noting that while sleep states are extremely useful, they cannot be used while the MCU is running an inference where high computational resources are required.

Providing a universally accepted power consumption measurement for a microcontroller is not an easy task, since there is not a universal set of rules available about the states and clock of core and peripherals during the measurement. While each MCU manufacturer provides the necessary tools and data to each developer to approximate an overall consumption per peripheral and clock usage for an application, that is of no use when it comes to ML models. This is mainly due to the fact that AI inference (i.e., runtime execution of ML models) relies heavily on mathematical calculations, memory throughput, data-bus traffic, and possible congestion events on top of application-specific peripheral requirements e.g., DSI interface for a camera in machine vision applications or SAI interface for microphones in voice recognition/commands applications. So, the problem of defining the “standard peripherals set for power measurement” arises when we need to provide real-world numbers.

To overcome these problems, we used the extremely popular and widely used Arduino Integrated Development Environment (IDE) as the development platform. The Arduino IDE is a software application that allows users to write, compile, and upload code to an Arduino microcontroller. It is based on the C language with a group of preset functions that are ready to use by the developer or user and may be used to manipulate a microcontroller’s peripherals and functions. The Arduino IDE includes a text editor for writing code, a built-in compiler for checking the code for errors, and a built-in serial monitor for communicating with the microcontroller. It also includes a variety of libraries that can be used to control the microcontroller’s peripherals and perform a wide range of tasks.

In our case, both the ESP32 and STM32 devices are supported, and most importantly, because of the preset functions existence requirement the Arduino IDE requires a minimum preset group of peripherals to be activated even when minimal code (blank main loop) is compiled and run. This requirement is set for every supported MCU and every architecture and it is implemented to hide the complexity of peripherals initialization from the end user. In our case, it is used as a standard initialization MCU state for generic usage. Thus, it provides a basis for a generic-usage basic power consumption state for each selected microcontroller.

Finally, no sleep or reduced power modes are applied throughout our code since power reduction gains are easy to calculate by the end developers if one wishes to do so. Moreover, usually low power states are not implemented while the core is running the ML model data. Thus, applying sleep modes is not of great significance in our case as they are finally presented as per inference power and only include inference power measurements.

### 3.3. Selected ML Models

For the results to have any meaningful impact and for our measurements to provide real-world results, the selection of models had to follow a group of criteria as follows:

- Each model is required to belong to a group of well-defined and well-known ML models. Specifically, all selected models should be popular models whose behavior is well known (their features and parameters are understood) or models that have been used in other scientific research in microcontroller-based systems [13,14,15]. This criterion will help to make our results meaningful and easier to understand.
- Each model should support at least one version of TensorFlow Micro Framework Operations to run in at least one of the selected MCUs with minimal alterations.
- Each model should be small enough to fit at least in one of the selected devices, preferably uncompressed, no external or added memory is permitted. This criterion maximizes the possibility that the results will contain pairs of compressed-uncompressed models on the same device to compare them while keeping the power measurements for the MCU unimpacted from any external device power consumption.
- The selected compression methods for a model should result in a smaller memory and/or model size footprint. While this criterion looks like a de-facto result of a compression application, this is not always true for ML models developed using the TensorFlow framework, e.g., pruning a model in TensorFlow framework will result in a more sparse model of the same storage size. To reduce its storage size a classical compression method may later be applied, such as gzip or a custom storage scheme with encoding [6]. The model in TensorFlow framework-based systems should be decompressed before it is usable for inference and this results in wasted computational time and power in the “realm” of microcontrollers. The final memory footprint remains the same and also more “temporary” storage is needed for the decompressed model. These facts defeat any generic usability of this method in TensorFlow Micro Framework.

To minimize any random discrepancies for each selected model the measurements were taken using random numbers as inputs of the model. This was done to minimize any case of favorable inputs (e.g., lots of zeros in multiplication).

#### 3.3.1. LeNet-5 Model

**Figure 5:**
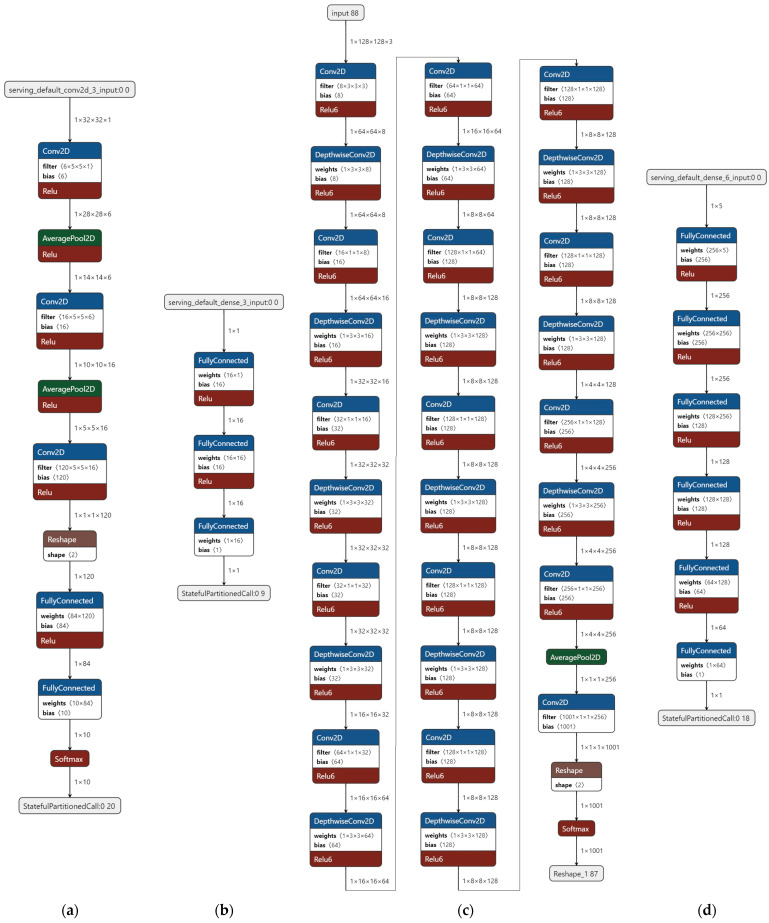
The selected Models. (**a**) LeNet-5, (**b**) Sine Calculator Model, (**c**) MobileNet-025, (**d**) IPS Model.

LeNet-5 (Figure 5a) is a convolutional neural network (CNN) architecture that was developed at AT&T Bell Labs in the 1990s [16]. It is a pioneering model in the field of deep learning, and it is often considered one of the first successful CNNs. The architecture of LeNet-5 consists of several layers of convolutional, pooling, and fully connected layers, which are designed to take raw image data as input and output a predicted class label for the input image. The model is named after the number of layers it contains (five convolutional and three fully connected layers), and it is commonly used for image classification tasks. This ML model is widely used with the MINST dataset for research purposes. This is a widely-used dataset for the benchmarking of image classification models. It contains a training set of 60,000 grayscale images of handwritten digits (0–9) and a test set of 10,000 images. The images are 28 × 28 pixels in size, and the task is to classify each image as belonging to one of the ten classes (i.e., the digits 0–9). LeNet-5 has been applied to the MNIST dataset, and it has been shown to achieve good performance on this dataset. In general, the MNIST dataset is considered to be a relatively simple dataset, and more complex datasets and tasks may require more powerful models than LeNet-5.

#### 3.3.2. Simple Sine Calculation Model

An ML model can be trained to predict the values of a sine wave using a dataset of sine wave samples. To train the model, the dataset would need to contain pairs of input and output values, where the input is a point on the x-axis of the sine wave, and the output is the corresponding y-value on the sine wave. This ML model (Figure 5b) could then be trained using a supervised learning algorithm to learn the relationship between the input and output values and make predictions about the y-value of a sine wave for a given x-value. Once the model has been trained, it could be used to predict the values of a sine wave for any x-value within the range of the training data. This in fact is a ready-to-use model included in TensorFlow Lite Framework, which is a version of TensorFlow Framework for embedded devices and Arduino IDE TinyML Library as a “Hello world” example. It is an extremely small model that provides some interesting results.

#### 3.3.3. MobileNet 025

MobileNet (Figure 5c) is a convolutional neural network (CNN) architecture that was designed by Google [17] for efficient on-device inference (Figure 5c). It is a lightweight model that is specifically optimized for use in applications on mobile devices. MobileNet uses depth-wise separable convolutions, which helps reduce the number of parameters in the model and makes it more efficient to run on mobile devices. MobileNet-025 is a variant of the MobileNet architecture that has a width multiplier of 0.25, which means that the number of filters in each layer is reduced by a factor of four compared to the base MobileNet architecture. This makes the model even more lightweight, efficient, and generally suitable for microcontroller-based devices, at the cost of some reduction in accuracy. In our case, this is the largest model we could fit to the selected devices.

#### 3.3.4. AI-Based Indoor Localization System

This model (Figure 5d) was developed for both mobile and microcontroller-based devices [14]. It runs successfully on real-world experiments and consists of five identical custom-made DNN Models that infer the distance of a device from nodes anchored to known positions within a residence or other indoor space. It measures the Received Signal Strength Indicator values (RSSI) of Bluetooth signals and feeds them to a trained model that rejects the artifacts of noise while increasing the precision of location calculation. Since all five models are identical, we only used one for our measurements.

### 3.4. Selected Framework

There are Frameworks that are built for ultra-low power applications such as those acknowledged in [18] that are focused on comparing two deep learning frameworks, QKeras and Larq, that support deep quantization for ultra-low precision machine learning. The authors are using these frameworks to develop and evaluate two applications: Human Activity Recognition and Anomaly Detection for Industry 4.0. The main advantage of using these frameworks for deep quantization is that they can enable the deployment of machine learning models on ultra-low power micro-controllers, which have applications in a variety of settings, including the Internet of Things (IoT) and Industry 4.0. The results of the comparison show that these frameworks can achieve good accuracy and inference time on ARM-based architectures, making them promising tools for ultra-low precision machine learning. However, it is also stated that these frameworks suffer from a lack of attention from the wider community and are thus less supported when comparing them to Frameworks like TinyML which is a variation of TensorFlow Micro.

While the attention of the community is of no consequence in our study, the real-world results are, especially the observations of various effects of compression in memory footprint/storage footprint versus the power efficiency per inference for different hardware architectures. Under this scope, it was decided that the framework we were going to use was TensorFlow Micro (or TinyML). TensorFlow Micro is a version of TensorFlow that is designed to be used on microcontrollers and other resource-constrained devices. It is a lightweight version of TensorFlow that is optimized for low-power and low-memory devices, and it allows developers to use TensorFlow’s machine-learning capabilities on these devices [19]. TensorFlow Micro is designed to be easy to use and integrate into existing applications, and it supports a range of hardware platforms and architectures, including ARM Cortex-M microcontrollers. With that said, even with this framework that is the most “mature” among all others, most widely used, and with better “stability” we noticed a lot of discrepancies that had to do mostly with version incompatibility and manufacturer support for TensorFlow Micro.

## 4. Measurements and Results

In this section, we present and analyze the results of our measurements. The TensorFlow version on ESP32 was 2.1.1 a rather old version that is lagging behind current development since there is no newer library available while the STM32 board was updated to version 2.5.0 but with the ability to run up to the latest version 2.11.0 if the developer uses the official STM32 development environment STM32CubeMX. Since TensorFlow Micro (or TinyML) only supports a subset of TensorFlow Lite operations and the differences between versions are, most of the time, not backward compatible. Not all models run on both devices without major modifications of their architecture and since any modification would have skewed the results we opted to measure and present the models that run in their original form.

**Figure 6:**
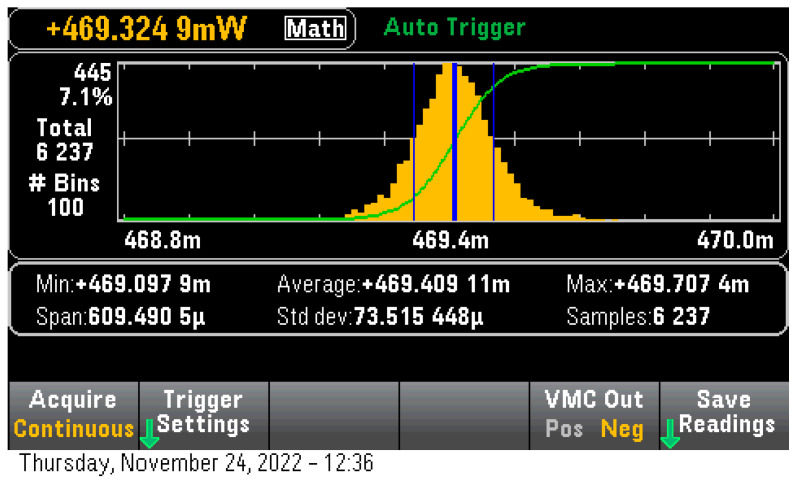
STM32H7 Idle consumption on Arduino Core.

To have a clearer view of each microcontroller’s power efficiency we measured the No-Operations loop power consumption with all the required Arduino peripherals enabled. We also left the default clocks unmodified, this meant that ESP32 run at 240 MHz on both cores and STM32 run at 400 MHz on its single core. ESP32 was found to run with 124.1 mW of power consumption, a rather typical consumption for this microcontroller with its WiFi and BlueTooth transceivers disabled while STM32 H7 was measured 469.4 mW (Figure 6). Since no inference model (or any other user code) was running no triggering event had to be set up, thus this measurement was taken in auto-triggering mode. The difference in idle power consumption was attributed to the large number of peripherals enabled on this device in its Arduino Core support library. The manufacturer indicated 263 uW per MHz for a total of 105.2 mW with all peripherals disabled.

The inference results were taken with the triggering mode enabled and the trigger for each measurement was generated in the code and signaled the starting of a new inference. The results include the total inference time for each device and each model, the power consumption for one inference, and the “pure power consumption” for an inference. The latter is calculated through the subtraction of idle power from the total power. “Pure power consumption” is a name given for this measurement and was selected as an indication of how efficient an MCU core is in running the mathematical model for inference. Real-world values are closer to inference power since pure power does not include any clocks, PLLs, or internal BUS consumption that are required for the core to function correctly.

Additionally, to make our measurement results more intuitive, we defined a theoretical “virtual battery” of 1000 mAh at 3.3 V as a basis to produce a “virtual total device runtime” on this battery. We set an inference rate for each model of three inferences per second (a period time of 333,333 uS for each inference) to accommodate for high latency models and consider any spare computing time as an MCU sleep state of zero power consumption. The “Virtual runtime” is based on the Total Power Per inference result for each model and device that includes any functional power consumption overhead for each device.

### 4.1. LeNet-5 Results

The model runs on both devices in uncompressed form but only runs in STM32 as a compressed model with Quantization Aware Training. This was due to the incompatibility of the older ESP32 TinyML OpCodes library with the compressed model. The initial size of the model was 245 kB and required 23 kB of Tensor Arena Size (basic memory footprint). The quantized model was 72 kB in size and required a tensor Arena size of 5.8 kB.

Inference time for the uncompressed model was measured to be 313,715 uS for the ESP32 and 19,393 uS for the STM32, more than 16 times faster, a fact that was attributed initially to the architecture of the ESP32 that uses an external Flash IC through Quad Serial Peripheral Interface (QSPI) to store both user code and model plus the slower clock of the ESP32.

**Figure 7:**
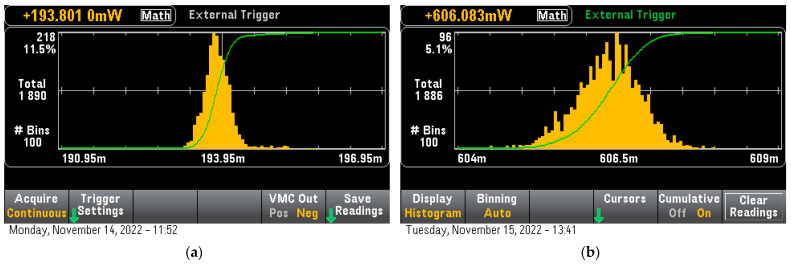
Uncompressed Lenet-5 Model Inference consumption (**a**) For ESP32, (**b**) For STM32H7.

Inference consumption for the ESP32 for the uncompressed model was 193.8 mW (Figure 7a) while for the STM32H7 was 606.6 mW (Figure 7b) taking into account the inference time needed for each device the total power per inference was calculated to be 16.888 uWh for the ESP32 and 3.26 uWh for the STM32H7. A big difference indicated was that the ARM core proved 5.2 times more power efficient in running math operations than the extended core, also taking into the use of low-throughput external storage on ESP32, which was hindering its core with wait-states that increased the overall power consumption.

Removing the idle-state consumption from the measurements gave a pure power rating of 6.08 uWh per inference for the ESP32 and 0.738 uWh per inference for the STM32H7. The STM32H7 was found to be 8.2 times more efficient in running ML models than the ESP32.

**Figure 8:**
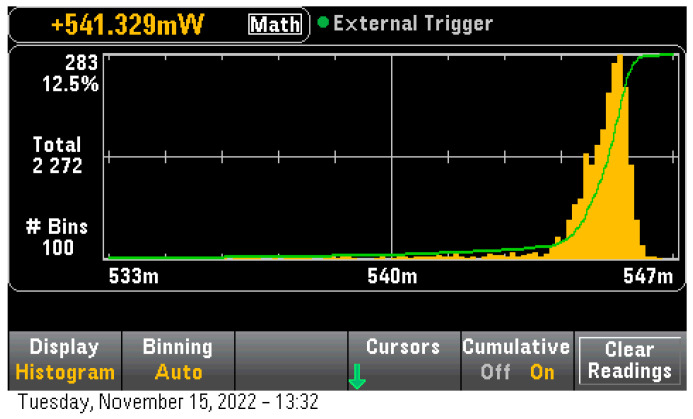
Quantized LeNet-5 Model Inference consumption on STM32H7.

On the compressed LeNet-5 on STM32H7 the total time per inference was found to be 6665 uS with a power consumption of 545.6 mW (Figure 8), this was an improvement in apparent power consumption of approximately 10%. However, considering the lower inference time the total required power per inference was calculated to be 1.01 uWh per inference an improvement of 3.2 times over the uncompressed power requirements. Finally, removing the idle power consumption gave us a 0.14 uWh per inference power consumption which was 5.2 times better than the uncompressed model on this device.

The uncompressed-to-compressed model size ratio was 3.4 and the memory footprint was about 4 times smaller for the quantized model. The pure power ratio was calculated to be around 5.3 times better for the compressed model and this difference was attributed not only to the lower required number of instructions and internal transfers throughput but also to the quantization effect that transformed part of the model into integer numbers limiting the usage of the Floating-Point Operations Unit (FPU) of the ARM core. So initially it looks like compressing a model improves its power efficiency in more than one way. Table 1, summarizes the results of Lenet-5.

### 4.2. Sine Model Results

**Figure 9:**
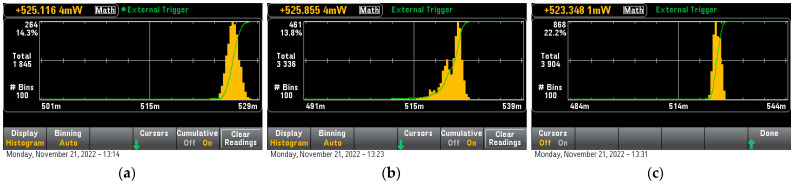
Sine Wave Prediction Model (**a**): Unoptimized, (**b**): Post Quantized, (**c**): Quantization Aware Trained.

This model run successfully only on STM32H7, in fact, it was possible to use two methods of quantization on it, both Quantization Aware (QA) training and Post Quantization (PQ). This model is extremely small and both compressed and uncompressed were 3 kB in size. Apparently, the framework’s model overhead was the major contributor to this model’s size. The tensor Arena size for the uncompressed model was 240 B, for the quantization-aware trained model was 144 B and for the post-quantized model was 120 B. The inference time for the uncompressed model was 13 uS, for the QA model was 12 uS and for the PQ model was 12.5 uS, we can consider them almost the same since they are within the measurement error margin. The inference power consumption for the non-optimized model was 526.2 mW and for the QA and PQ models was 525.2 mW and 524.8 mW respectively (Figure 9). The calculated per inference power for the non-optimized, QA, and PQ models was 0.0019 uWh, 0.0017 uMh, and 0.0018 uWh respectively, all within 5% of each other respectively. Finally, removing the idle state power from the total inference power resulted in 0.0002 uWh, 0.00018 uWh, and 0.00019 uWh for the non-optimized, the PQ, and the QA models respectively. All the measurements were consistent indicating that QA models are more power efficient than PQ models. The similarity of the results, shown on Table 2, was attributed to the fact that the models were much smaller than the size of the data cache of the MCU, so it is entirely possible that they were running from this level of hierarchical memory totally bypassing any internal BUS transfers or wait states.

### 4.3. MobileNet-025 Model Results

This model was bigger than others; in its uncompressed state it has a 1.84 MB size and the equivalent QA model is 497 k. Non-optimized, it requires more than 512 kB of SRAM to run (4 bytes more in fact), and quantized it requires 128 kB of SRAM. Since no external memory is permitted, this model runs only on the STM32H7 device and only in its quantized form. While STM32H7 has 1 MB of SRAM available it only offers 512 kB of SRAM as continuous memory, the other 512 kB are chunks or TCM and other SRAM spread across three different address spaces. It was therefore impossible (by 4 bytes) to load the model.

As for the ESP32, the available SRAM was not enough to run any form of this model, and while this device supports external PSRAM (PseudoStatic RAM) and there was a board available with 4 MB of PSRAM connected over through the same QSPI BUS as the Flash memory the results would have been extremely skewed by the usage of the external memory especially on the same BUS as the Storage Unit.

**Figure 10:**
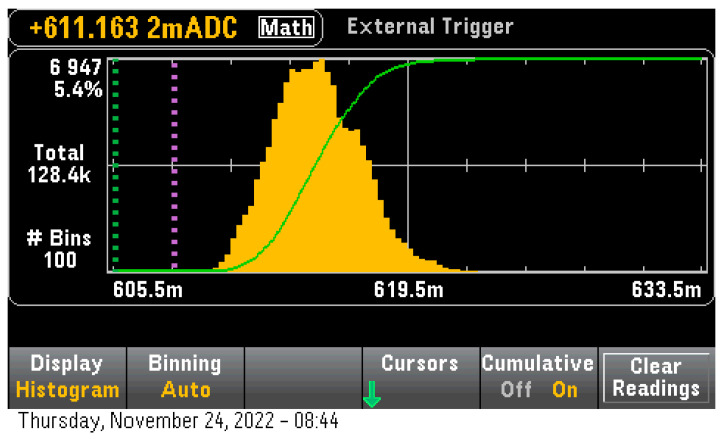
MobileNet-025 Inference Power consumption.

The measurements for the PQ model on STM32H7 resulted in an inference time of 464,400 uS with an inference power consumption of 615.3 mW (Figure 10). This led to the calculation of 79.37 uWh power consumption per inference and 18.82 uWh per inference pure power consumption.

Comparing this model with the Lenet-5 we can see that the size was approximately 7 times bigger (72 kB vs 497 kB). However, the per inference pure power consumption was more than 100 times bigger (0.14 uWh/inference to 18.82 uWh/inference) and almost 80 times bigger as a total power per inference. We also noticed that the TensorFlow Arena size was far greater on the MobileNet-025, 128 kB vs 5.8 kB (approximately 22 times more SRAM required). Therefore, it is entirely possible that the required memory (SRAM) size of a model is affecting its overall power efficiency far more than its total model size that fits the available storage. This is even more evident when we keep in mind that both SRAM and Flash are constantly under use since the full model cannot fit the SRAM (in contrast to mobile devices, higher resource capable Raspberry-PIs, and Personal Computers). Results for this model are gathered in Table 3.

### 4.4. BlueTooth IPS Model Results

**Figure 11:**
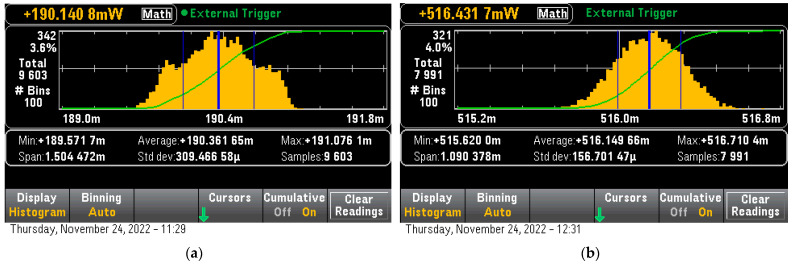
Uncompressed IPS Model Inference consumption (**a**) For ESP32, (**b**) For STM32H7.

This model is included as a large (for a microcontroller) custom DNN model with a total size of 492 kB, however, it differed from the rest of the large models by the fact that it requires a very small Tensor Arena size to run, with approximately 3 kB required. Unfortunately, as the custom DNN model does not support some of the OpCodes at least not without heavily modifying it- it was only possible to run on both devices as an uncompressed model. The inference time was 24,850 uS for the ESP32 and 2624 uS for the STM32H7 with an inference power consumption of 190.3 mW and 516.2 mW respectively (Figure 11). The calculated power per inference result was 1.31 uWh/inference for the ESP32 and 0.376 uWh/inference for the STM32H7 while for the pure power per inference, the result was 0.457 uWh/inference and 0.034 uWh/inference respectively.

Comparing these results with the results of the uncompressed LeNet-5 model we find that the ESP32 consumes approximately 13 times more energy per inference for the LeNet-5 model although it is smaller in overall size. Again, the size of the SRAM used by the model (TensorFlow Arena 23 kB for the LeNet vs 3 kB for the IPS model) affects the power consumption of the model significantly more that the overall size. The same observations are found in the STM32H7 device with 8.68 times and 21.65 times more power used for the total power per inference and pure power per inference respectively when running the LeNet-5 Model that requires more SRAM. Results for this model are organized in Table 4.

## 5. Conclusions, Discussion, and Future Work

In this paper, we used state-of-the-art compression methods on well-known ML models under strict rules to observe their overall performance in terms of power efficiency. We also compared both the compressed and uncompressed characteristics of the ML models to observe any power efficiency effects related to them. The results indicated that the power consumption of ML models on microcontrollers is affected by much more characteristics than their mere size of them.

Almost all the research work reviewed in Section 2, approaches the problem of power efficiency mostly from the view of memory footprint reduction. The results of these studies show that the gains in power consumption are linearly affected by the reduction in the size of the ML model after applying any optimization methods. There are some excellent methods proposed that approach this problem in a more “classical” way, assigning states to the algorithm and preventing the microcontroller, microprocessor, or TPU from constantly running the inference model resulting in improved power consumption over time. Most of the research is based on systems with medium to high resources available, such as multi-core ARM microprocessors and TPUs presenting overall power results for each device in constant running inference over different models, with few exceptions that take into account microcontroller-based systems but are not presenting any accurate power measurement results.

While the results of previous works are extremely useful for developers and researchers that wish to build efficient models for microcontrollers with small memory footprints, they don’t really offer too much insight into what to expect from a power-consumption aspect for an autonomous system after applying different methods of optimization. Thus, this study that presents the specific effects of power optimization of ML models running on MCUs and identifies some possible aspects that a designer may take into account when designing such systems, may prove to be useful. This study presents experimental measurements, producing hard facts for known ML models and widely available MCU architectures providing useful insight with respect to the relation of MCU architectural features and power-efficient ML model execution.

Although the hardware differences from device to device make it extremely difficult to measure the power requirements unless strict rules are applied, to minimize external effects that may skew the results, we managed to draw some initial conclusions.

SRAM usage of an ML model is a much better indication of power efficiency than the overall size of the model. We observed larger models running on systems with less sophisticated Flash storage and overcome-energy wise-this deficiency in comparison to smaller models that used more SRAM and run on a system with a better Flash storage architecture.

Applying compression to a model, other than decreasing its storage size, may:

- Decrease the requirements in SRAM that leads to much lower deployment requirements and more efficient power usage
- Decrease the usage of core subsystems (such as hardware FPU) that leads to a wider pool of selectable hardware per application (non-hardware-FPU capable units) and increase power efficiency during inference calculations of ML models.
- Increase the cache hit ratio of the microcontrollers memory system leading to a reduction of internal data traffic and increasing the overall power efficiency

Further work is required in a much larger set of models to systematically quantify our findings. It could also be useful to include, in future work, more frameworks for our measurements to solidify our conclusions.

## Figures and Tables

**Table 1:** Lenet-5 Results.

Device	Model	Optimization	Inference Time (uS)	Inference Power (mW)	NoOp Power	SRAM (Tensor Arena Size)	Model Size	Total Power Per Inference (uWh)	Pure Inference Power (uWh)	Virtual Runtime (h)
ESP32	Lenet-5	NO	313,715	193.8	124	23 k	245 k	16.88832417	6.082585278	18.09
STM32H7	Lenet-5	QA	6665	545.6	469.4	5.8 k	72 k	1.010117778	0.141075833	302.49
STM32H7	Lenet-5	NO	19,393	606.5	469.4	23 k	245 k	3.267181806	0.738550083	93.52

NO: Not Optimized, QA: Quantization Aware Training.

**Table 2:** Sine Model Results.

Device	Model	Optimization	Inference Time (uS)	Inference Power (mW)	NoOp Power	SRAM (Tensor Arena Size)	Model Size	Total Power Per Inference (uWh)	Pure Inference Power (uWh)	Virtual Runtime (h)
STM32H7	Sine	NO	13	526.2	469.4	240	3 k	0.001900167	0.000205111	160,804
STM32H7	Sine	QA	12	524.6	469.4	144	3 k	0.001748667	0.000184	174,736
STM32H7	Sine	PQ	12.5	524.8	469.4	120	3 k	0.001822222	0.000192361	167,683

NO: Not Optimized, QA: Quantization Aware Training, PQ: Post Quantized.

**Table 3:** MobileNet-025 Model Results.

Device	Model	Optimization	Inference Time (uS)	Inference Power (mW)	NoOp Power	SRAM (Tensor Arena Size)	Model Size	Total Power Per Inference (uWh)	Pure Inference Power (uWh)	Virtual Runtime (h)
STM32H7	MobileNet 025	PQ	464,400	615.3	469.4	128 K	497 k	79.3737	18.8211	3849

**Table 4:** BlueTooth IPS Model Results.

Device	Model	Optimization	Inference Time (uS)	Inference Power (mW)	NoOp Power	SRAM (Tensor Arena Size)	Model Size	Total Power Per Inference (uWh)	Pure Inference Power (uWh)	Virtual Runtime (h)
ESP32	BT-IPS	No	24,850	190.3	124	3 k	492 k	1.313598611	0.457654167	232.6
STM32H7	BT-IPS	No	2624	516.2	469.4	3 k	492 k	0.376252444	0.034112	812.1

## Data Availability

No new data were created or analyzed in this study. Data sharing is not applicable to this article.
